# Supplementary material for: Electronically phase separated nano-network in antiferromagnetic insulating LaMnO3/PrMnO3/CaMnO3 tricolor superlattice
Source: Nat Commun. 2022 Nov 3;13:6593. doi: 10.1038/s41467-022-34377-4 (PMC9633694; doi:10.1038/s41467-022-34377-4)
Supplement: Supplementary file 1 — Supplementary Information [file 41467_2022_34377_MOESM1_ESM.pdf]

Supplementary Materials
Electronically phase separated nano-network in antiferromagnetic insulating $LaMnO_3/PrMnO_3/CaMnO_3$ tricolor superlattice

Qiang Li[1,2]*, Tian Miao[2,3]*, Huimin Zhang[2,4,5]*, Weiyan Lin[1], Wenhao He[1,2], Yang Zhong[2,4,5], Lifen Xiang[2], Lina Deng[2], Biying Ye[2], Qian Shi[2], Yinyan Zhu[1,5,6], Hangwen Guo[1,5,6], Wenbin Wang[1,5,6], Changlin Zheng[1,2], Lifeng Yin[1,2,5,6,7,8], Xiaodong Zhou[1,5,6 †], Hongjun Xiang[2,4,5 †] and Jian Shen[1,2,5,6,7,8†]

[1]State Key Laboratory of Surface Physics and Institute for Nanoelectronic Devices and Quantum Computing, Fudan University, Shanghai 200433, China
[2]Department of Physics, Fudan University, Shanghai 200433, China
[3]School of Materials Science and Engineering, Xi'an Jiaotong University, Xi'an, Shanxi 710049, China
[4]Key Laboratory of Computational Physical Sciences (Ministry of Education) and Institute of Computational Physical Sciences, Fudan University, Shanghai 200433, China
[5]Shanghai Qi Zhi Institute, Shanghai 200232, China
[6]Zhangjiang Fudan International Innovation Center, Fudan University, Shanghai 201210, China
[7]Shanghai Research Center for Quantum Sciences, Shanghai 201315, China
[8]Collaborative Innovation Center of Advanced Microstructures, Nanjing 210093, China

[*]These authors contributed equally to this work
[†]Emails: zhouxd@fudan.edu.cn, hxiang@fudan.edu.cn, shenj5494@fudan.edu.cn

Contents:

A: Basic structural and physical properties characterization

B: Additional TEM characterizations of the superlattice

C: Working principles of scanning microwave impedance microscopy

D: Additional MFM and sMIM imaging of nano-network in another sample

E: Absence of nano-network in tricolor superlattice film grown on $NdGaO_3$ substrate

F: Additional DFT results

G: Manipulation of nano-network EPS state via tip mechanical perturbation

Figure S1 to S12

SI A: Basic structural and physical properties characterization

We conduct basic structural and physical properties characterization of the 60 nm tricolor superlattice thin film discussed in the main text to assess its sample quality. Figure S1 shows the

structural properties. We use *in situ* reflection high-energy electron diffraction (RHEED) to monitor the thin film growth process. The intensity oscillation in RHEED (Fig. S1b) indicates the alternating unit-cell by unit-cell growth of the $LaMnO_3$ (red), $PrMnO_3$ (yellow) and $CaMnO_3$ (blue) layers. The superlattice displays an atomic flat surface with the corrugation less than 1 nm (Fig. S1a). Note that such AFM image was taken at room temperature in the as-grown state (before the first cool-down), which doesn't show any nanoscale network of structure protrusions, in contrast to Fig. 1b of the main text. It is another evidence that the nano-network is closely linked to the $SrTiO_3$ (STO) structural transition at low temperatures.

We also conduct a careful X-ray diffraction (XRD) measurement to determine the strain state of superlattice. Figure S1c presents the XRD reciprocal space map (RSM) collected around (103) reflection peak of the superlattice film grown on STO substrate. The epitaxial growth relationship is clearly confirmed in such RSM, i.e., the in-plane lattice constant of the superlattice film is locked to the STO substrate. The lattice constant of superlattice film can be further determined in XRD by performing the 2θ scan along different lattice planes. Figure S1d shows the XRD obtained around (002), (012) and (112) peaks. According to the Bragg equation $2dsin\theta = n\lambda$ and the association rules between Miller indices and the interplanar spacing, the relation between crystal lattice constant and diffraction peak is established as the follow $\frac{1}{d_{hkl}^2} = \frac{h^2}{a^2} + \frac{k^2}{b^2} + \frac{l^2}{c^2}$. We take the STO cubic lattice constant 3.905Å and the XRD in Fig. S1d as three known relative relationships between STO and superlattice to calculate the three lattice constants of superlattice as $a = 3.902$Å, $b = 3.904$Å and $c = 3.772$Å. Given the lattice constant of bulk $(La_{1-y}Pr_y)_{1-x}Ca_xMnO_3$ is smaller than 3.905Å, XRD results unambiguously demonstrate that the superlattice film on the STO substrate is tensile strained resulting in the extended in-plane lattice constants and compressed out-of-plane lattice constant.

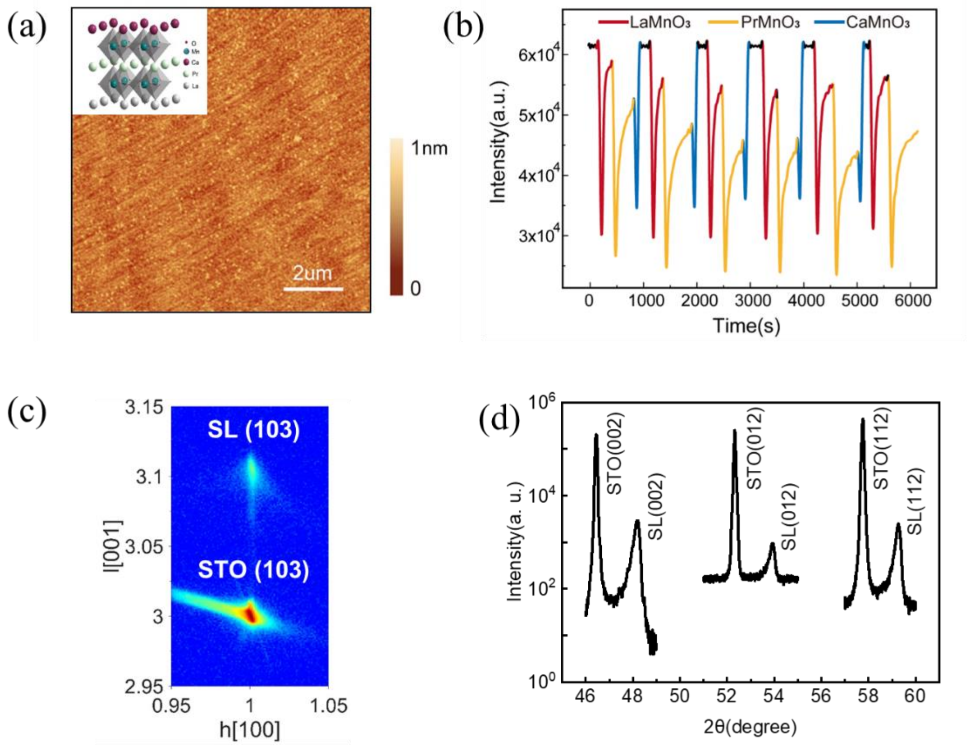

Fig. S1. Growth and crystal structure of $LaMnO_3$/$PrMnO_3$/$CaMnO_3$ tricolor superlattice on

STO (001) substrate. a AFM image of the as-grown superlattice film. The crystal structure of such fully A-site ordered tricolor superlattice is shown in the inset. **b** RHEED intensity oscillation represents the layer-by-layer growth process from different targets. **c** The XRD reciprocal map around (103) peak of the superlattice film on STO substrate. **d** XRD scan around (002), (012) and (112) peaks of the superlattice film on STO substrate.

We measure the macroscopic transport and magnetic properties of the superlattice using Quantum Design Physical Property Measurement System (PPMS) and Quantum Design superconducting quantum interference device system (SQUID), respectively. Figure S2a displays the resistance versus temperature at different magnetic fields. The film is in an insulating state with almost no thermal hysteresis up to 9 T, indicating the absence of electronic phase separation in such a tricolor superlattice similar to previous reports. Figure S2b shows the magnetization as a function of temperature. Interestingly, such M-T behavior is not expected from an antiferromagnetic insulating phase. Nor is the typical behavior of a ferromagnetic (FM) metallic phase. It appears that this sample has a FM contribution that doesn't form a long-range order, which inspires us to further explore it under microscopic scales.

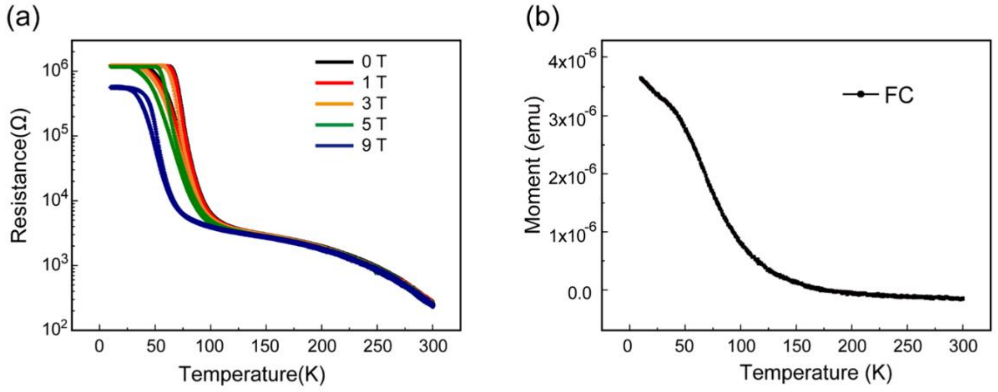

Fig. S2. Transport and magnetic measurements of 60 nm tricolor superlattice on STO (001) substrate. a Temperature-dependent resistance (*R*-*T*) measurement at different magnetic fields. **b** Temperature-dependent magnetization curve (*M*-*T*).

SI B: Additional TEM characterizations of the superlattice

(1) structural dislocations at the nano-network domain wall.

We performed a focus series high angle annular dark-field scanning transmission electron microscopy (HAADF-STEM) imaging (Fig. S3) of the domain wall with the in-plane view sample. When the probe focus changed (Fig. S3 b-d), shifted lattice planes emerged within the domain wall. This is a clear evidence that there is a dislocation underneath the surface of the domain walls. The dislocation is captured with the optical sectioning of the incoherent aberration corrected HAADF-STEM with enhanced depth resolution, which is similar to the phenomena observed by H. Yang and P. Nellist *et.al*[1].

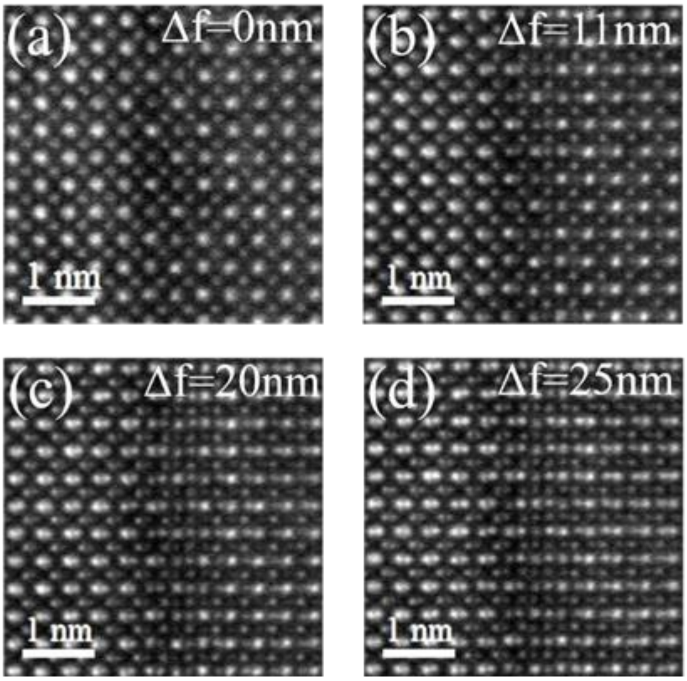

Fig. S3. Focus series HAADF-STEM imaging of the domain wall.

(2) The structure and chemistry of the tricolor superlattice film.

We perform both XRD and TEM to characterize the tricolor superlattice structure of the LPCMO film. The $LaMnO_3$/$PrMnO_3$/$CaMnO_3$ superlattice structure of our film is best evidenced by the XRD measurement showing the existence of all superlattice peaks (Fig. S4a). The atomic structure of the superlattice was also examined in cross-sectional view (along the [110] zone axis) using HAADF-STEM imaging, as shown Fig. S4b. The intensity line profile along the growth direction clearly resolves the alternating growth of the Pr, La and Ca layers, according to the z-contrast mechanism of HAADF imaging.

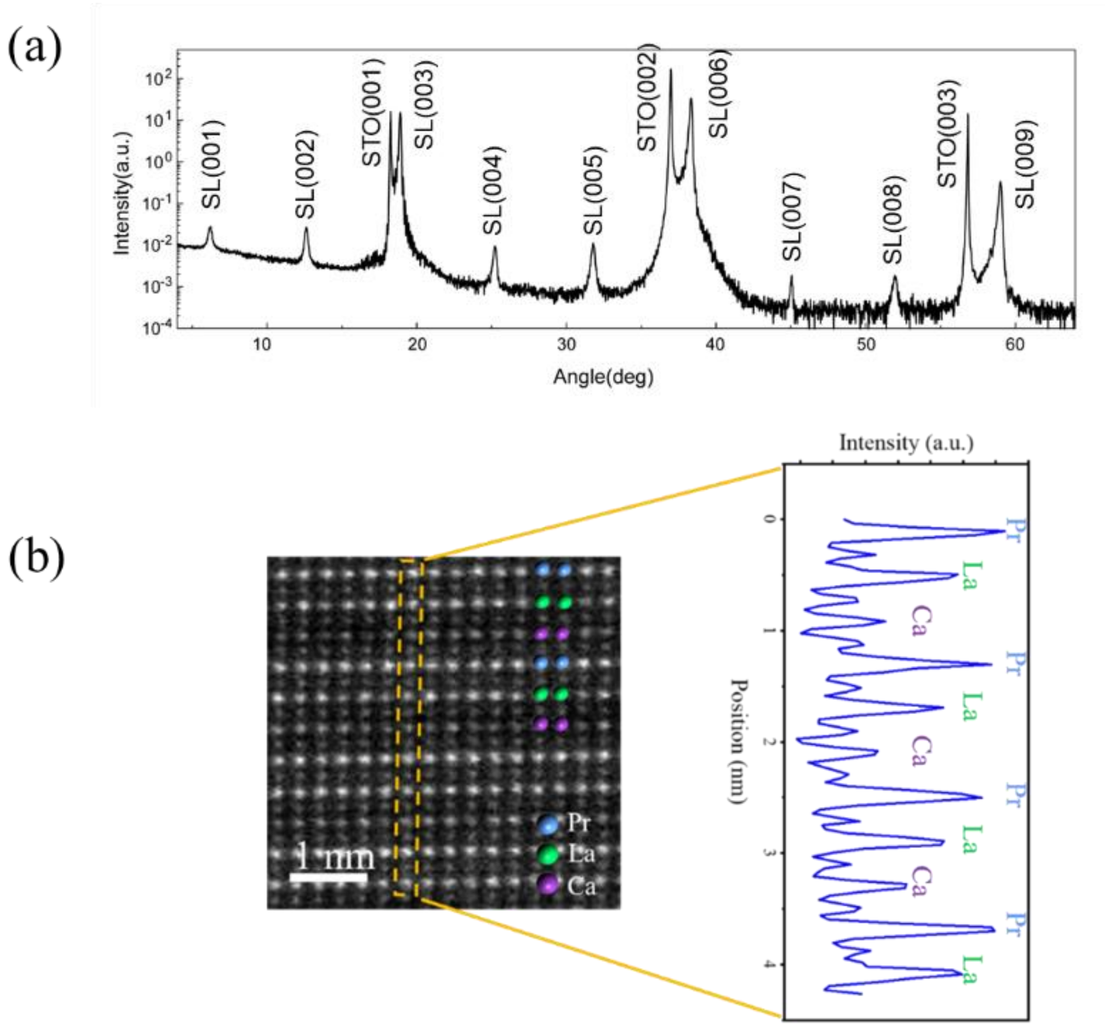

Fig. S4. The structure and chemistry of the tricolor superlattice film. a The XRD measurement shows superlattice peaks. **b** HAADF-STEM image in [110] zone axis. HAADF intensity line profile along the growth direction clearly shows the alternating growth of the Pr, La and Ca layers.

(3) The chemistry of the nano-network domain wall.

The chemical composition of the domain walls (in-plane view) was analyzed using STEM electron energy loss spectroscopy (STEM-EELS). The EELS spectra were recorded with a Gatan Continuum HR/1066 energy filter system with 100 pA beam current. The semi-convergent angle of the probe forming lens is 21.4 mrad, and the semi-collection angle of the GIF entrance aperture is 38 mrad. The window 2 in the HAADF image of Fig S5a shows the location of the domain wall, while window 1 and 3 show the surrounded matrix. The integrated EELS spectra from the three regions are compared in Fig. S5b. No significant elemental difference could be found within the domain wall respect to the surrounded area.

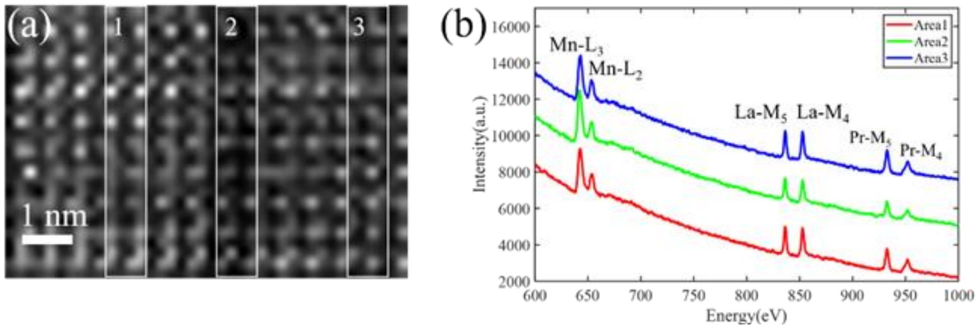

Fig. S5. STEM-EELS analysis of the domain wall with in-plane view. a HAADF-STEM image of the domain wall (marked with the white window 2) and the surrounded matrix (window 1 and 3). **b** Integrated EELS spectra from the marked three regions in Fig S5a.

(4) Strain analysis along the domain wall.

The STEM strain analysis was also performed along the directions which are parallel to the domain walls (DWs) in Fig. 1d and e of the main text. Figure S6a shows the relative change of the averaged lattice spacing along the [010] direction in 0° DW, while Fig. S6b shows the same plot but along [110] direction in 45° DW. In both measurements, no significant strains were found in the directions parallel to the DWs, which indicates that the compressive strain is uniaxial in nature.

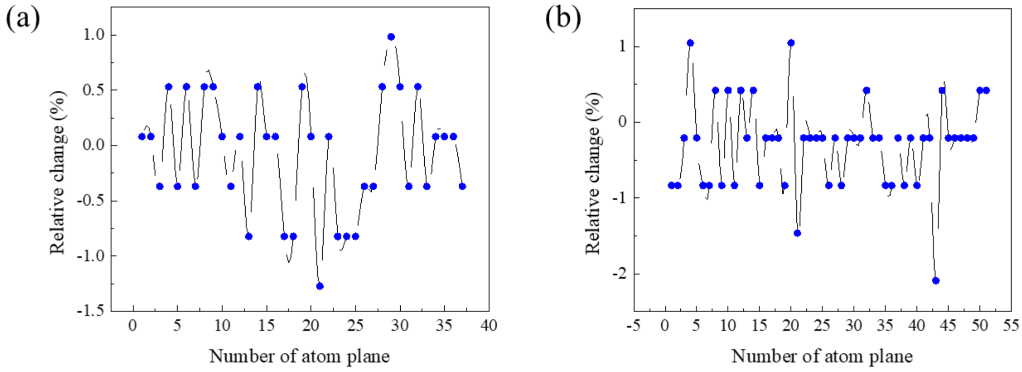

Fig. S6. STEM strain analysis along the directions parallel to the domain walls. a Relative change of the averaged lattice spacing along the [010] direction in 0° domain wall. **b** Relative change of the averaged lattice spacing along the [110] direction in 45° domain wall.

SI C: Working principles of scanning microwave impedance microscopy

Scanning microwave impedance microscopy (sMIM) is a recently developed scanning probe microscopy for nanoscale conductivity imaging. It can be taken as a near-field scanning optical microscopy working at the microwave frequency. Figure S7a shows a schematic of sMIM experimental setup. A 3 GHz microwave signal is delivered to an atomic force microscope tip apex and the reflected microwave signal is collected and demodulated into two output signal sMIM-Im and sMIM-Re, which are proportional to the imaginary and real components of the tip-sample admittance, respectively. This tip-sample admittance is solely determined by the spatial profile of

the complex dielectric constant $\hat{\varepsilon}(r) = \varepsilon'(r) + i\left[\varepsilon''(r) + \sigma(r)/\omega\right]$ for the entire region around the tip-sample interface. In our experiment, it is the sample's local variation of conductivity $\sigma(r)$ that affects the tip-sample admittance and gives rise to the sMIM imaging contrast.

The dependence of sMIM signal on sample's local conductivity can be simulated by using a commercial finite element analysis (FEA) software COMSOL. Figure S7b shows the result of such FEA simulation which is also called sMIM response curve. As one can see, the sMIM-Im signal has a monotonic dependence on the sample's local conductivity (represented by the sheet resistance here) while sMIM-Re doesn't, i.e., a higher sMIM-Im signal suggests a larger local conductivity. Therefore, the sMIM-Im signal is used throughout the work to characterize the local conductivity.

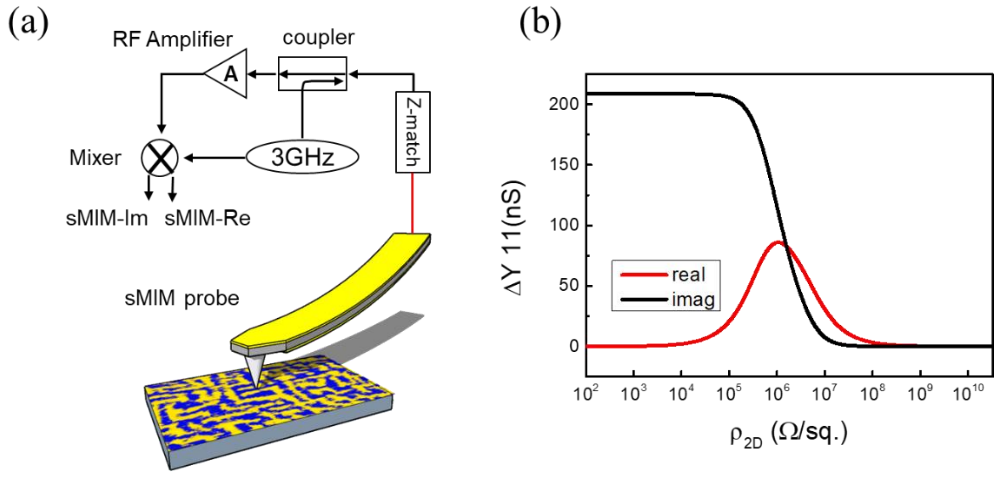

Fig. S7. Working principles of sMIM. a The schematic of sMIM experimental setup **b** The sMIM response curve shows the dependence of tip-sample admittance on the sheet resistance of the thin film.

SI D: Additional MFM and sMIM imaging of nano-network in another sample

We provide additional AFM, MFM and sMIM data taken on another superlattice sample grown on STO substrate to demonstrate the repeatability of our observation of nano-network electronic phase separation (EPS). Figure S8a and b are MFM images of the same area taken at 12 K and 200 K, respectively. The nano-network EPS pattern is clearly seen and persists above the STO structural phase transition temperature 105 K. Note that the used color scale of MFM image at 200 K is one order of magnitude smaller than that of 12 K. Shown in Fig. S8d is the sMIM measurement conducted on the same area at 2 K (red square in Fig. S8b). This measurement was done in another temperature cycle in which the same EPS pattern is observed as well. It not only demonstrates that the ferromagnetic phase at nano-network regions is metallic, but also reaffirms the persistence of such EPS pattern against thermal cycle. The simultaneously taken AFM image is presented in Fig. S8c in which the structural protrusion of EPS network can be resolved.

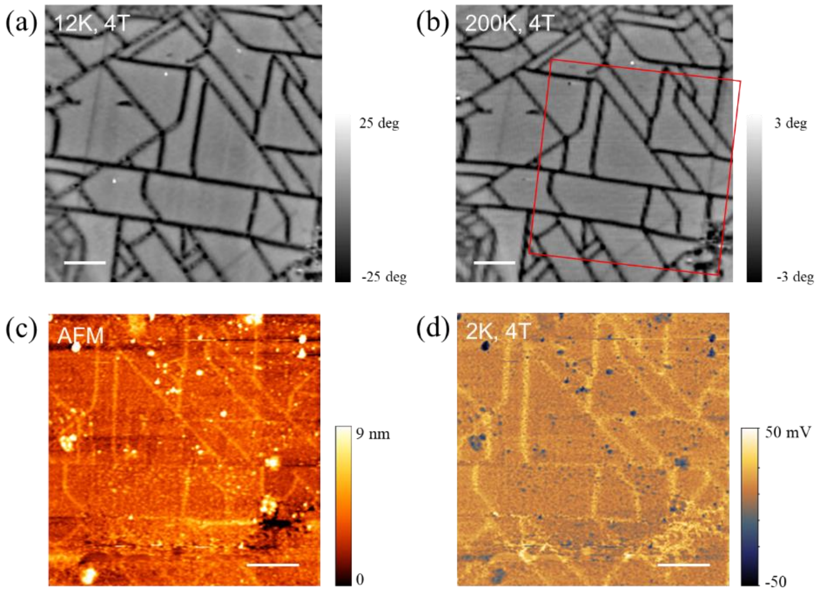

Fig. S8. Scanning probe imaging of EPS nano-network. a-b MFM images of EPS nano-network taken at 12 K and 200 K, respectively. **c** AFM image of the red square area denoted in Fig. S8b. **d** sMIM image of the red square area denoted in Fig. S8b. Scale bar is 2 μm.

SI E: Absence of nano-network in tricolor superlattice film grown on $NdGaO_3$ substrate

The same tricolor superlattice film has been grown on $NdGaO_3$ (NGO) substrate which doesn't have a structural phase transition at low temperatures. Therefore, the superlattice film remains in a single uniform antiferromagnetic insulating phase upon temperature cycling. We have reported this observation in our previous work[2]. Here we show some low temperature (10 K) field dependent MFM images reproduced from that work to demonstrate the absence of nano-network in superlattice film grown on NGO substrate. The red circles in Fig. S9 denote the defect areas in MFM imaging which serve as a marker to make sure all field dependent MFM images were taken from the same sample area.

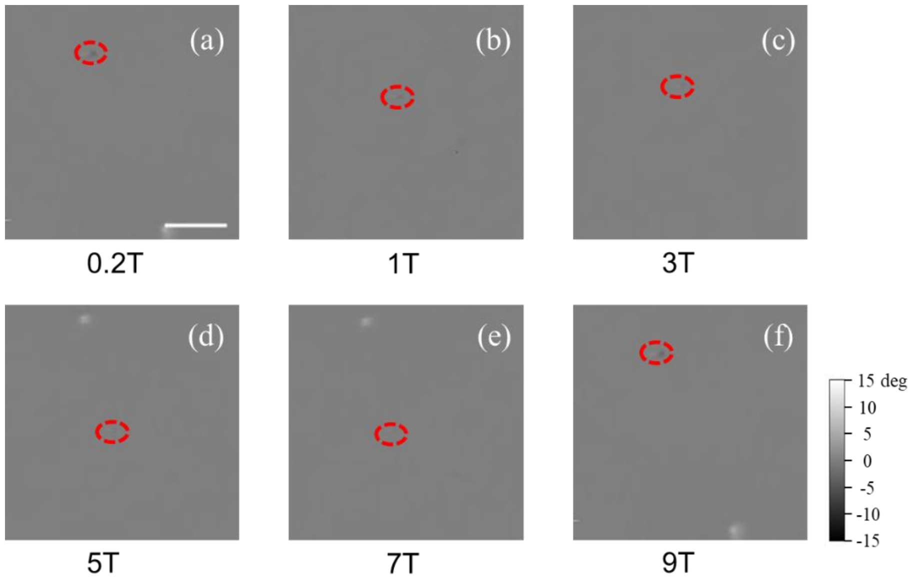

Fig. S9. MFM images of superlattice film on NdGaO3 substrate. a-f Field dependent MFM images taken at 10 K. Scale bar is 2 μm.

SI F: Additional DFT results

For searching the structural ground state, a series of distorted candidate superlattice structures are generated by the perturbation on an initial undistorted LPCMO superlattice. The lattice parameters of the initial undistorted LPCMO superlattice are randomly oscillated by 10% and the coordinates of each atom were randomly shifted by up to 0.3 Å. Structural optimization was performed on these candidate structures and several structures with lower energy are obtained. They are listed below with the space group number and tilting mode. Energies are calculated using DFT method. The one with $a^{-}a^{-}c^{+}$ tilting mode and space group number 33 has the lowest energy which implies it is the most stable structure of LPCMO tricolor superlattice film.

No.	Group No.	Tilting mode	E (meV)/Mn
1	33	$a^{-}a^{-}c^{+}$	0.00
2	7	$a^{-}a^{-}c^{-}$	6.03
3	33	$a^{-}a^{-}c^{-}$	33.10
4	46	$a^{-}a^{-}c^{0}$	34.12
5	36	$a^{-}a^{-}c^{-}$	95.81
6	29	$a^{-}a^{-}c^{-}$	96.45

Table S1. The energy difference of possible octahedral tilting modes. The energy of $a^{-}a^{-}c^{+}$ mode is set to be the reference.

The uniaxial strain is also checked by only constraining the lattice a of LPCMO on the STO substrate. In Fig. S10, it displays the same magnetic phase transition phenomenon with the situation of biaxial strain that the phase of A-AFM can transit to FM phase when the lattice parameter becomes shorter. However, the critical lattice constant for the phase transition in the biaxial strain

case is smaller. Note that the predicted critical lattice constant from the DFT calculations may not exactly correspond to the experimental value.

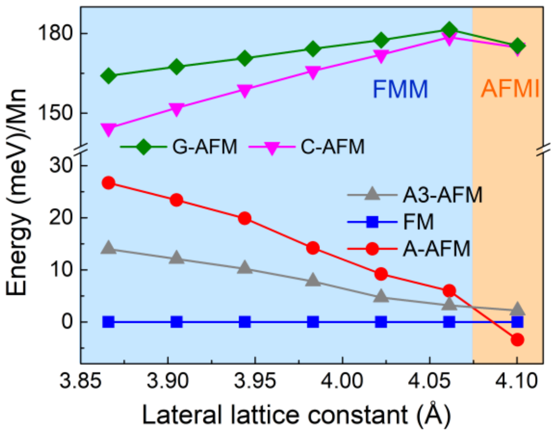

Fig. S10. Uniaxial strain analysis. The energies of different magnetic states varying with different lattice parameters *a* of STO while the lattice parameter *b* is kept to be 3.905Å. The energy of FM state is set as a reference for energy comparison.

LPCMO film is stacked with A site atom La, Pr, Ca and Mn-O octahedron and fixed on the STO substrate. The distortion of octahedron in LPCMO can be influenced when the lattice of substrate changes. One AFM phase (*a*=*b*=4.10 Å) and one FM phase (*a*=*b*=3.905 Å) are chosen for analyzing the octahedron distortion. These six bond lengths in three different Mn-O octahedrons (Mn1-O, Mn2-O and Mn3-O) are plotted in Fig. S11. The difference of bond length in one octahedron reveals the degree of Jahn-Teller distortion. Clearly, FM state has a less distortion than AFM state.

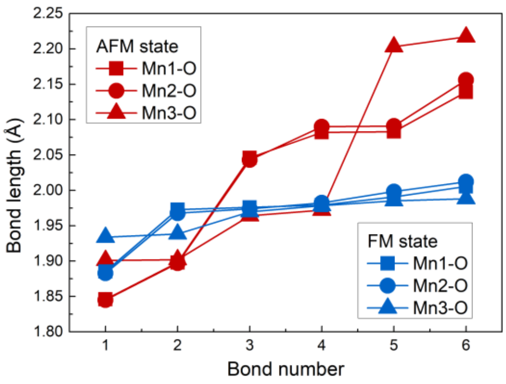

Fig. S11. Jahn-Teller distortion analysis. The bond lengths of six Mn-O bonds in Mn1-O, Mn2-O and Mn3-O octahedrons, respectively which are arranged according to the magnitude of bond length. For the considered AFM state, the lattice parameters *a*=*b*=4.10 Å are adopted, whereas the lattice parameters are set to be *a*=*b*=3.905 Å for the FM state.

SI G: Manipulation of nano-network EPS state via tip mechanical perturbation

The tricolor superlattice film grown on STO substrate is in a critical tensile strained state and therefore highly sensitive to the local strain perturbation. In addition to the STO DWs induced strain

engineering, here we show preliminary results of manipulating nano-network EPS state via a tip mechanical perturbation. In particular, we use MFM tip to apply a local force to the film to change its strain state, and expect a similar local response from the film as it does to the STO DWs formation. Figure S12a is a MFM image taken at 12 K and 8 T to show the nano-network EPS state as reported in the main text. We then scan the film in a contact mode along the red line denoted in Fig. S12a, but with a compressive load almost three times of the load in a normal scan. After such tip pressing, we re-image the same area in a normal load to obtain Fig. S12b. Very interestingly, a dark stripe now appears at the red line position indicating the formation of a ferromagnetic metallic phase. This observation strongly indicates that, the nano-network EPS state found in our tricolor superlattice is indeed subjected to an active manipulation via local strain which is essential for its applications.

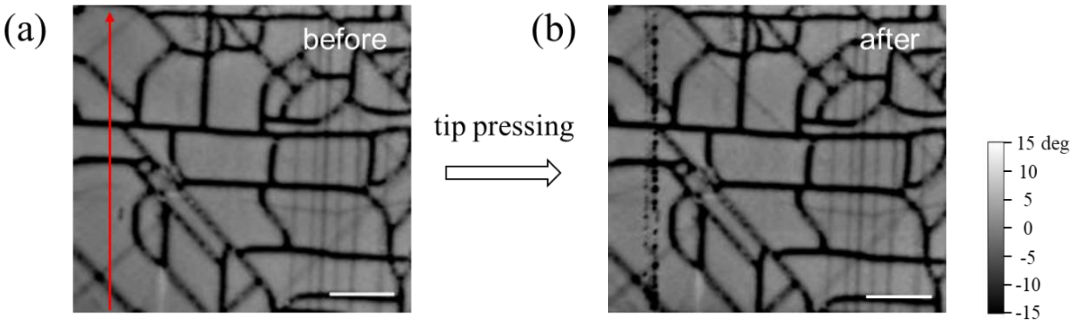

Fig. S12. Tip manipulation of nano-network EPS state. The MFM images taken at 12 K and 8 T before (a) and after (b) the tip pressing along the red line denoted in the image. Scale bar is 2 μm.

Supplementary References

1 Yang, H. *et al.* Imaging screw dislocations at atomic resolution by aberration-corrected electron optical sectioning. *Nat. Commun.* **6**, 7266, (2015).

2 Miao, T. *et al.* Direct experimental evidence of physical origin of electronic phase separation in manganites. *Proc. Natl. Acad. Sci. U.S.A.* **117**, 7090-7094, (2020).
